# Abnormal myosin post‐translational modifications and ATP turnover time associated with human congenital myopathy‐related *RYR1* mutations

**DOI:** 10.1111/apha.14035

**Published:** 2023-08-21

**Authors:** Alexander Sonne, Anna Katarina Antonovic, Elise Melhedegaard, Fariha Akter, Jesper L. Andersen, Heinz Jungbluth, Nanna Witting, John Vissing, Edmar Zanoteli, Arianna Fornili, Julien Ochala

**Affiliations:** ^1^ Department of Biomedical Sciences, Faculty of Health and Medical Sciences University of Copenhagen Copenhagen Denmark; ^2^ Department of Chemistry, School of Physical and Chemical Sciences Queen Mary University of London London UK; ^3^ Department of Orthopaedic Surgery, Institute of Sports Medicine Copenhagen Copenhagen University Hospital, Bispebjerg and Frederiksberg Copenhagen Denmark; ^4^ Center for Healthy Aging, Department of Clinical Medicine University of Copenhagen Copenhagen Denmark; ^5^ Department of Paediatric Neurology Evelina London Children's Hospital London UK; ^6^ Randall Centre for Cell and Molecular Biophysics, Muscle Signalling Section, Faculty of Life Sciences and Medicine King's College London London UK; ^7^ Copenhagen Neuromuscular Center, Department of Neurology University of Copenhagen Copenhagen Denmark; ^8^ Departamento de Neurologia, Faculdade de Medicina, Hospital das Clínicas Universidade de São Paulo São Paulo Brazil

**Keywords:** acetylation, ATP, congenital myopathy, myosin heavy chain, phosphorylation, relaxed state, skeletal muscle

## Abstract

**Aim:**

Conditions related to mutations in the gene encoding the skeletal muscle ryanodine receptor 1 (*RYR1*) are genetic muscle disorders and include congenital myopathies with permanent weakness, as well as episodic phenotypes such as rhabdomyolysis/myalgia. Although RYR1 dysfunction is the primary mechanism in *RYR1*‐related disorders, other downstream pathogenic events are less well understood and may include a secondary remodeling of major contractile proteins. Hence, in the present study, we aimed to investigate whether congenital myopathy‐related *RYR1* mutations alter the regulation of the most abundant contractile protein, myosin.

**Methods:**

We used skeletal muscle tissues from five patients with *RYR1*‐related congenital myopathy and compared those with five controls and five patients with *RYR1*‐related rhabdomyolysis/myalgia. We then defined post‐translational modifications on myosin heavy chains (MyHCs) using LC/MS. In parallel, we determined myosin relaxed states using Mant‐ATP chase experiments and performed molecular dynamics (MD) simulations.

**Results:**

LC/MS revealed two additional phosphorylations (Thr1309‐P and Ser1362‐P) and one acetylation (Lys1410‐Ac) on the β/slow MyHC of patients with congenital myopathy. This method also identified six acetylations that were lacking on MyHC type IIa of these patients (Lys35‐Ac, Lys663‐Ac, Lys763‐Ac, Lys1171‐Ac, Lys1360‐Ac, and Lys1733‐Ac). MD simulations suggest that modifying myosin Ser1362 impacts the protein structure and dynamics. Finally, Mant‐ATP chase experiments showed a faster ATP turnover time of myosin heads in the disordered–relaxed conformation.

**Conclusions:**

Altogether, our results suggest that *RYR1* mutations have secondary negative consequences on myosin structure and function, likely contributing to the congenital myopathic phenotype.

## 1 INTRODUCTION

Mutations in the ryanodine receptor 1 (*RYR1*) gene lead to a wide spectrum of congenital myopathies such as central core disease (CCD), multi‐minicore disease (MmD), centronuclear myopathy (CNM), and congenital fibre type disproportion (CFTD), with an overall estimated prevalence of 1 in 90 000.^1^, ^2^, ^3^ *RYR1* mutations can also induce malignant hyperthermia (MH), a potentially life‐threatening pharmacogenetic reaction to volatile anesthetics and muscle relaxants, as well as exertional rhabdomyolysis and myalgia (ERM).^2^, ^3^, ^4^ A continuum between *RYR1*‐related MH and ERM is increasingly recognized.^5^ Finally, *RYR1* mutations have been linked to non‐skeletal muscle manifestations including a mild bleeding disorder due to smooth muscle dysfunction.^6^ The precise molecular and cellular mechanisms by which various *RYR1* mutations directly or indirectly promote downstream pathogenic events remain incompletely understood.

The *RYR1* gene encodes an essential Ca^2+^ release channel RyR1, located in the sarcoplasmic reticulum of skeletal muscles.^7^ Most of the *RYR1* mutations associated with CCD are dominant and missense, substituting one residue in the RyR1 sequence and causing not only a sarcoplasmic reticulum Ca^2+^ leak at rest^8^ but also a disruption of the Ca^2+^ transport through pores.^9^ Mutations related to MmD, CNM, and CFTD are typically recessive, often compound heterozygous with at least one truncating variant, lowering the protein expression of RyR1 and of other related sarcoplasmic reticulum proteins, thereby negatively impacting excitation–contraction coupling.^8^ Despite a good understanding of the principal underlying mechanisms, the precise pathophysiology is currently far from being fully identified and likely to be complex with potential involvement of mutation‐independent mechanisms and/or proteins beyond the sarcoplasmic reticulum. There is currently no cure for any *RYR1*‐related disorders although disease‐modifying approaches have been investigated or are currently in development.^10^

A number of probably under‐appreciated pathological modifications secondary to a mutated RyR1 receptor have previously been reviewed.^8^ For instance, transgenic mice expressing a point mutation at position 522 on the RyR1 molecule (Tyr to Ser), a model of the human *RYR1* MH mutation Y522S, display a disproportionate Ca^2+^ leak together with damaged and enlarged mitochondria due to secondary nitrosylation alterations.^11^ Thus, changes in the amount of post‐translational modifications (PTMs), including nitrosylation, as well as oxidation, glutathionylation, acetylation, phosphorylation, and palmitoylation, may thus fine‐regulate RyR1 and/or related contractile proteins.^8^, ^12^ In addition to RyR1, skeletal muscle requires the proper functioning of the sarcomere, in particular of its most abundant protein, myosin. Myosin molecules have a turnover rate of 1%–2% per day, making them preferential targets for different types of damaging PTMs.^13^, ^14^, ^15^ Hence, in the present study, we hypothesized that, in *RYR1‐*related congenital myopathies, unusual PTMs in particular acetylation and phosphorylation would appear on myosin molecules, more specifically on its myosin heavy chains (MyHCs) rich in lysine, serine, tyrosine, and threonine residues.

To allow proper muscle functioning, MyHCs have numerous conformations.^7^ In resting skeletal muscle, there is an energy‐saving super‐relaxed state where myosin heads are anchored to the filament backbone. There is also an ATP‐consuming disordered–relaxed state where myosin heads are not blocked and have an ATPase activity up to 10 times higher than in the super‐relaxed state.^16^, ^17^ These specific states can be disrupted by changes in the levels of PTMs, especially, those involving acetylation and phosphorylation.^18^ Thus, in the present study, we further hypothesized that in congenital myopathies due to *RYR1* mutations, aberrant PTMs on MyHCs would be associated with an increase in the proportion of myosin molecules in the disordered–relaxed state, thereby impairing the ATP demand of resting myofibres and indirectly explaining some of the myopathic phenotypes.

To test our hypotheses, we used muscle biopsy samples from five patients with congenital myopathy due to *RYR1* mutations and compared them with five age‐ and gender‐matched controls and with five patients with *RYR1* mutations and rhabdomyolysis/myalgia but without muscle weakness.^19^ We then determined the nature and location of PTMs using LC/MS together with an assessment of myosin super‐relaxed/disordered–relaxed states using Mant‐ATP chase experiments in isolated membrane‐permeabilized muscle fibres.

## 2 RESULTS

### 2.1 Specific PTMs on β/slow and type IIa MyHCs in the presence of myopathy‐linked *RYR1* mutations

**FIGURE 1:**
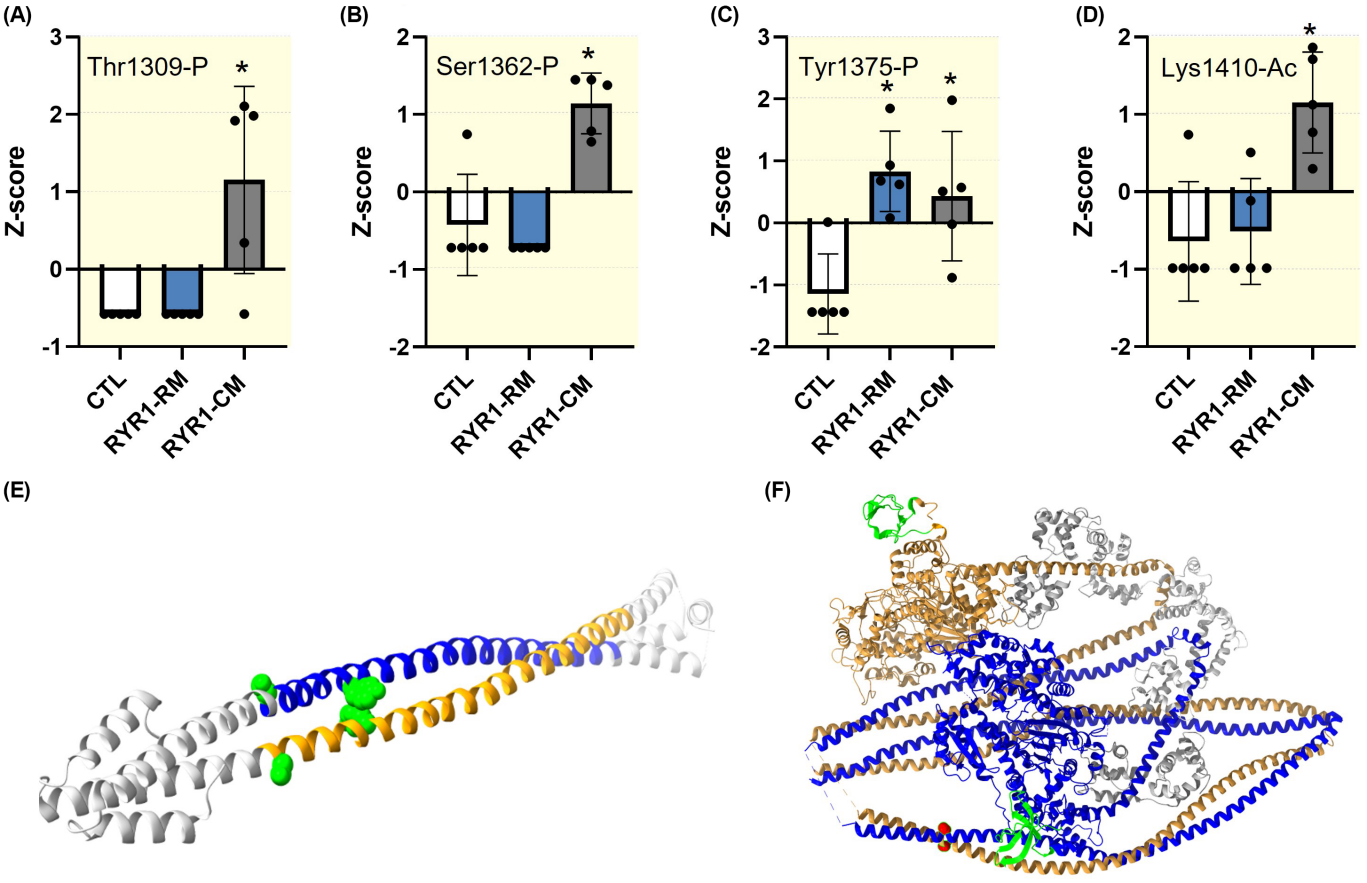
Disrupted β/slow myosin heavy chain (MyHC) post‐translational modifications. (A–D) depict four significant differences observed in acetylation and phosphorylation. They show the *z*‐score for each post‐translational modification. Dots are individual subject's average data. Means and standard deviations also appear on histograms. *denotes a difference (*p* < 0.05) between controls (CTL) and *RYR1* patients (RYR1‐CM) or patients with *RYR1* mutations and rhabdomyolysis/myalgia but without any weakness (RYR1‐RM). (E) represents myosin II skip 2 (RCSB PDB:4XA3) with Ser1362‐P and Tyr1375‐P in green. (F) is the myosin II dimer in its closed conformation (10S myosin II, RCSB PDB:6XE9 aligned with MYH7 using BLAST). The SH3 domain is shown in green, while myosin regulatory and essential light chains are displayed in gray. Lys1410‐Ac appears in red.

**FIGURE 2:**
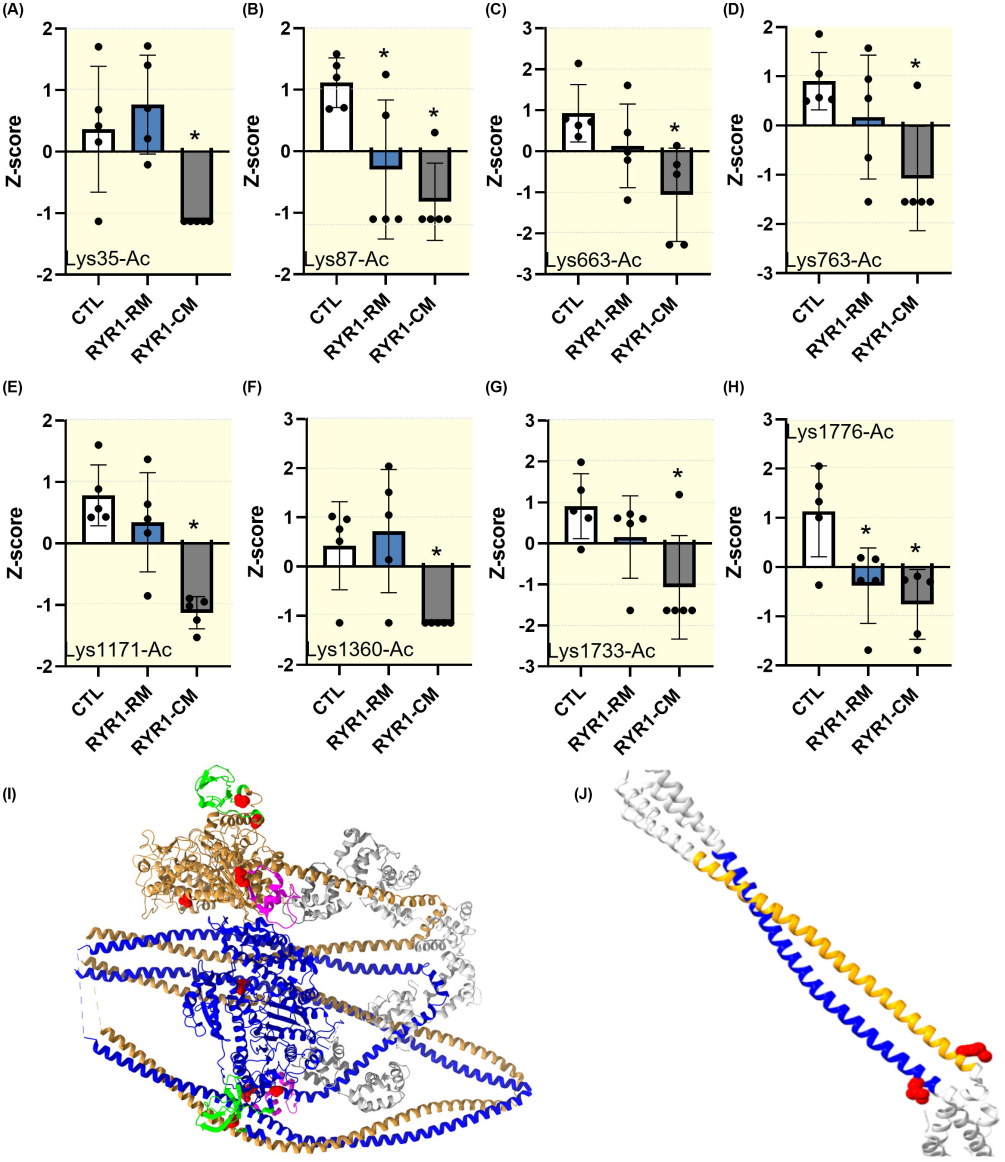
Abnormal post‐translational modifications on type IIa myosin heavy chain (MyHC). (A–H) show the eight significant differences found in acetylation and phosphorylation. They depict the *z*‐score for each acetylation. Dots are individual subject's average data. Means and standard deviations also appear on histograms. *denotes a difference (*p* < 0.05) between controls (CTL) and RYR1 patients (RYR1‐CM) or patients with *RYR1* mutations and rhabdomyolysis/myalgia but without any weakness (RYR1‐RM). (I) is the myosin II dimer in its closed conformation (10S myosin II, RCSB PDB:6XE9 aligned with MYH2 using BLAST). The SH3 domain is in green, myosin regulatory and essential light chains are in gray, and the converter region is in pink. Lys35‐Ac, Lys87‐Ac, Lys663‐Ac, and Lys763‐Ac are represented in red. (J) is a schematic view of myosin II skip 1 (RCSB PDB: 4XA1) with Lys1171‐Ac in green.

A total of five patients with *RYR1* mutations and diagnosed with a congenital myopathy were compared to five age‐ and gender‐matched controls, as well as five patients with *RYR1* variants but without weakness (Table 1). Muscle biopsy specimens were run on 6% SDS–PAGE for MyHC isoform separations and determination.^20^ β/slow (P12883—MYH7_HUMAN) and type IIa MyHC (Q9UKX2—MYH2_HUMAN) isoform gel bands were excised and screened for the most common PTMs, that is, acetylation and phosphorylation. We then normalized the peptide site intensities to the maximum peak intensities for both β/slow and type IIa MyHCs for each of the 15 human individuals. We further calculated the mean ± standard deviation for each group and converted the intensities to *z*‐scores. Nine myopathy‐associated PTMs were identified. Two additional aberrant phosphorylations and one new acetylation were observed on the tail region of the β/slow MyHC of myopathic patients, specifically, that is, Thr1309‐P, Ser1362‐P, and Lys1410‐Ac (Figure 1A–F). Additionally, six acetylations were missing on the type IIa MyHC of patients with congenital myopathy. These were located over the whole length of the type IIa MyHC, that is, Lys35‐Ac, Lys663‐Ac, Lys763‐Ac, Lys1171‐Ac, Lys1360‐Ac, and Lys1733‐Ac (Figure 2A–E). Besides these myopathy‐linked PTMs, we also found one phosphorylation, Tyr1375‐P, that was on the β/slow MyHC of all the *RYR1* patients (myopathic individuals and those suffering from exertional rhabdomyolysis/myalgia syndrome; Figure 1A–F). We finally observed two acetylations lacking, Lys87‐Ac and Lys1776‐Ac, on the type IIa MyHC of all the individuals carrying *RYR1* mutations (Figure 2A–E).

**TABLE 1:** Patient and control muscle biopsy samples used.

Group	Age at biopsy (years)	Gender (M/F)	*RYR1* mutation(s)	Conditions
RYR1‐RM	32	M	c.7300G>A^a^	Adult‐onset rhabdomyolysis/myalgia syndrome and malignant hyperthermia susceptibility
RYR1‐RM	33	M	c.14545G>A^a^	Adult‐onset rhabdomyolysis/myalgia syndrome and malignant hyperthermia susceptibility
RYR1‐RM	45	F	c.1840C>T^a^	Adult‐onset rhabdomyolysis/myalgia syndrome and malignant hyperthermia susceptibility
RYR1‐RM	38	M	c.6617C>T^a^	Adult‐onset rhabdomyolysis/myalgia syndrome
RYR1‐RM	34	F	c. 6502G>A^a^	Adult‐onset rhabdomyolysis/myalgia syndrome and malignant hyperthermia susceptibility
RYR1‐CM	23	F	c.14422‐14423TT>AA	Congenital myopathy with central cores
RYR1‐CM	22	M	c.718N>T and c.2897N>T	Congenital myopathy (congenital fibre type disproportion)
RYR1‐CM	22	M	c.13891T>C	Congenital myopathy with central cores and rods
RYR1‐CM	30	F	c.14818G>C	Congenital myopathy with central cores and rods
RYR1‐CM	13	M	c.9611C>T and c.14545G>A^a^	Congenital myopathy (centronuclear myopathy) with many internalized nuclei
CTL	22	M	–	–
CTL	34	F	–	–
CTL	51	F	–	–
CTL	41	M	–	–
CTL	27	F	–	–

^a^ Indicates that the specific *RYR1* mutation is listed on https://www.emhg.org/ because of its malignant hyperthermia susceptibility.

### 2.2 Altered ATP turnover rate of disordered–relaxed myosin heads in the presence of myopathy‐related *RYR1* mutations

**FIGURE 3:**
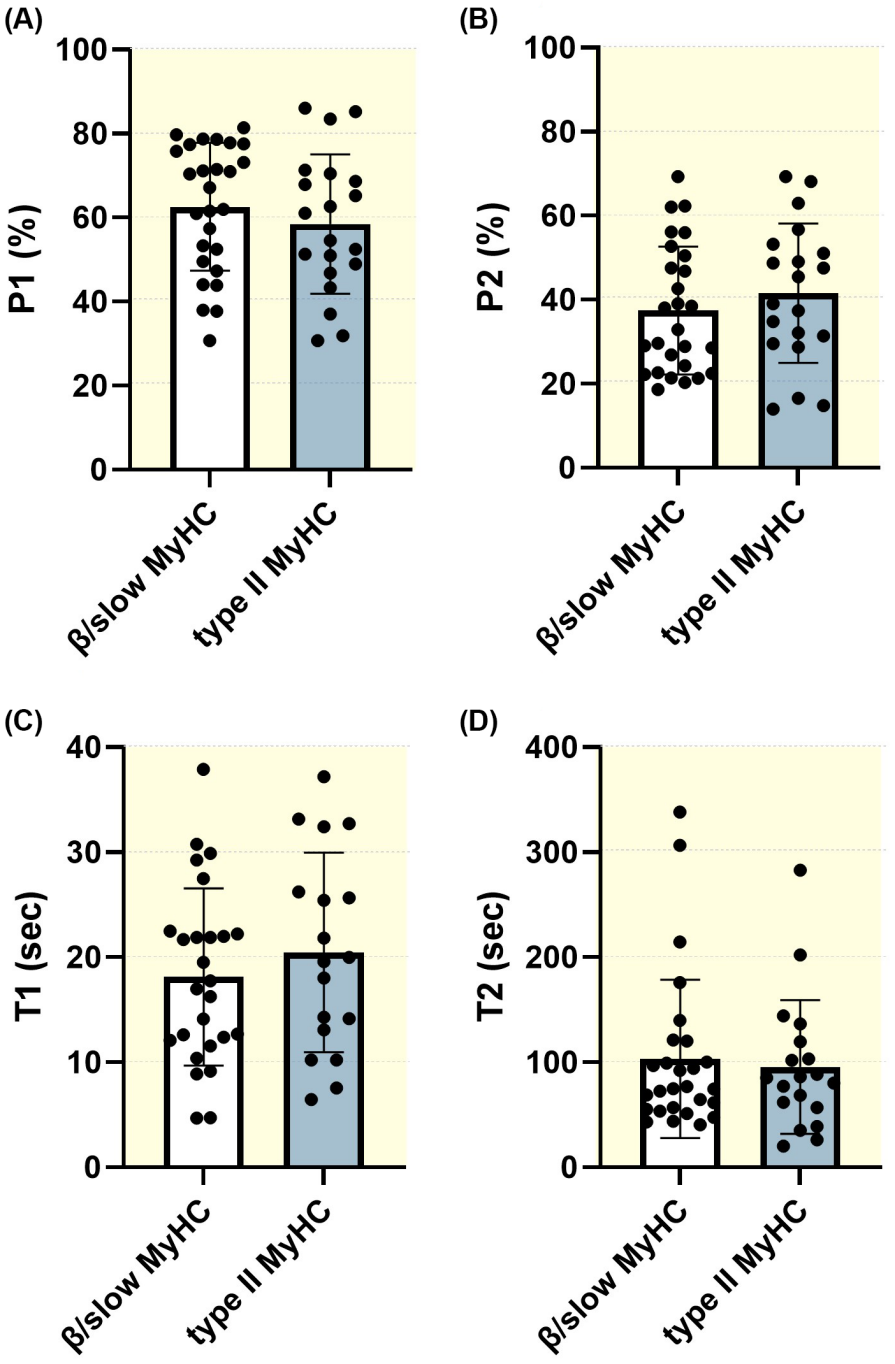
No effect of myosin heavy chain types (MyHCs) on proportions of myosin relaxed states and their ATP turnover lifetime time. The proportions of myosin heads in the disordered–relaxed (A, P1) and super‐relaxed states (B, P2), as well as their respective ATP turnover lifetimes (C, T1 and D, T2), are depicted. Dots are individual control muscle fibres' data. Means and standard deviations for each fibre type (β/slow or type II MyHC) also appear on histograms.

**FIGURE 4:**
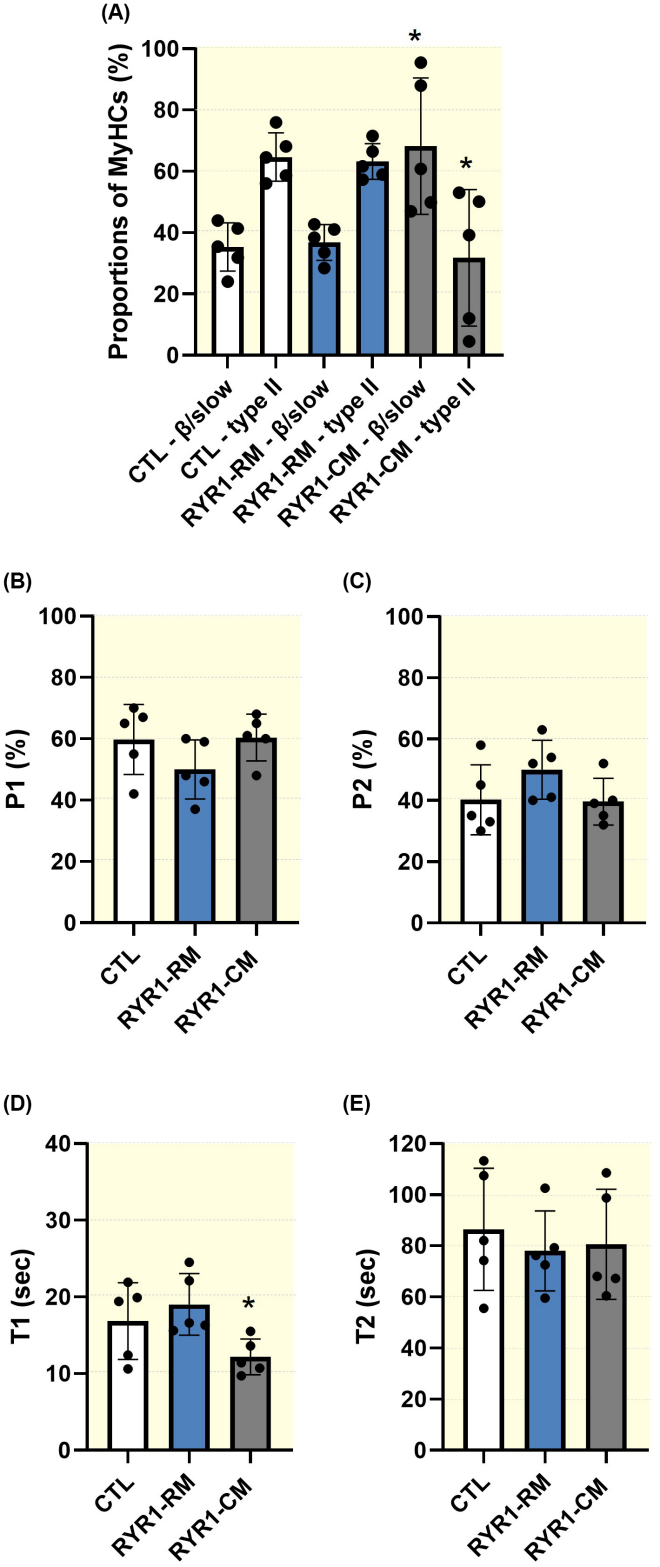
Increased ATP turnover lifetime of myosin disordered–relaxed state. (A) The proportions of β/slow and type II myosin heavy chain (MyHC) isoforms for controls (CTL), *RYR1* myopathic patients (RYR1‐CM), and patients with *RYR1* mutations and rhabdomyolysis/myalgia but without any weakness (RYR1‐RM) are presented. The proportions of myosin heads in the disordered–relaxed (B, P1) and super‐relaxed states (C, P2), as well as their respective ATP turnover lifetimes (D, T1 and E, T2), are shown. Dots are individual subject's average data. Means and standard deviations for each subject also appear on histograms. *denotes a difference (*p* < 0.05) between RYR1‐CM and CTL or RYR1‐RM.

To assess whether the presence or absence of aberrant phosphorylation/acetylation would impact myosin functionality, we evaluated the proportions of myosin heads in the super‐relaxed and disordered–relaxed states using the Mant‐ATP chase protocol.^21^ This method assumes that the myosin ATPase activity is up to 10 times higher in the disordered–relaxed state than in the super‐relaxed state. Hence, monitoring the decay in the fluorescence of Mant‐ATP over time allows the estimation of the proportion of myosin molecules in both states and respective ATP turnover rates.^21^ A total of 217 muscle fibres from the 15 controls and patients were mounted for Mant‐ATP chase experiments. The number of myofibres tested varied between individuals as some of the biopsy specimens from the myopathic patients with *RYR1* mutations were difficult to handle, that is, between 6 and 22 fibres mounted per subject. As we did not observe any significant MyHC‐specific differences in the proportions of myosin molecules in the disordered–relaxed state (P1), in super‐relaxed (P2), or in their ATP turnover lifetimes, T1 and T2 (Figure 3A–D), we pooled all the β/slow and type II MyHC fibres together for each subject (note that the various MyHC proportions based on the isolated muscle fibres are presented in Figure 4A). Our results then showed no effects of *RYR1* mutations on P1 and P2 (Figure 4B,C). However, the ATP turnover lifetime of the disordered–relaxed myosin heads (T1) was significantly decreased in myopathic patients when compared to controls and non‐myopathic *RYR1* patients (Figure 4D,E). Importantly, this implies that the rate of ATP hydrolysis of myosin heads in the disordered–relaxed state from myopathic individuals gets faster independently of fibre (MyHC) types.

### 2.3 Disrupted myosin rod structure and dynamics with the addition of phosphorylation on residues

**FIGURE 5:**
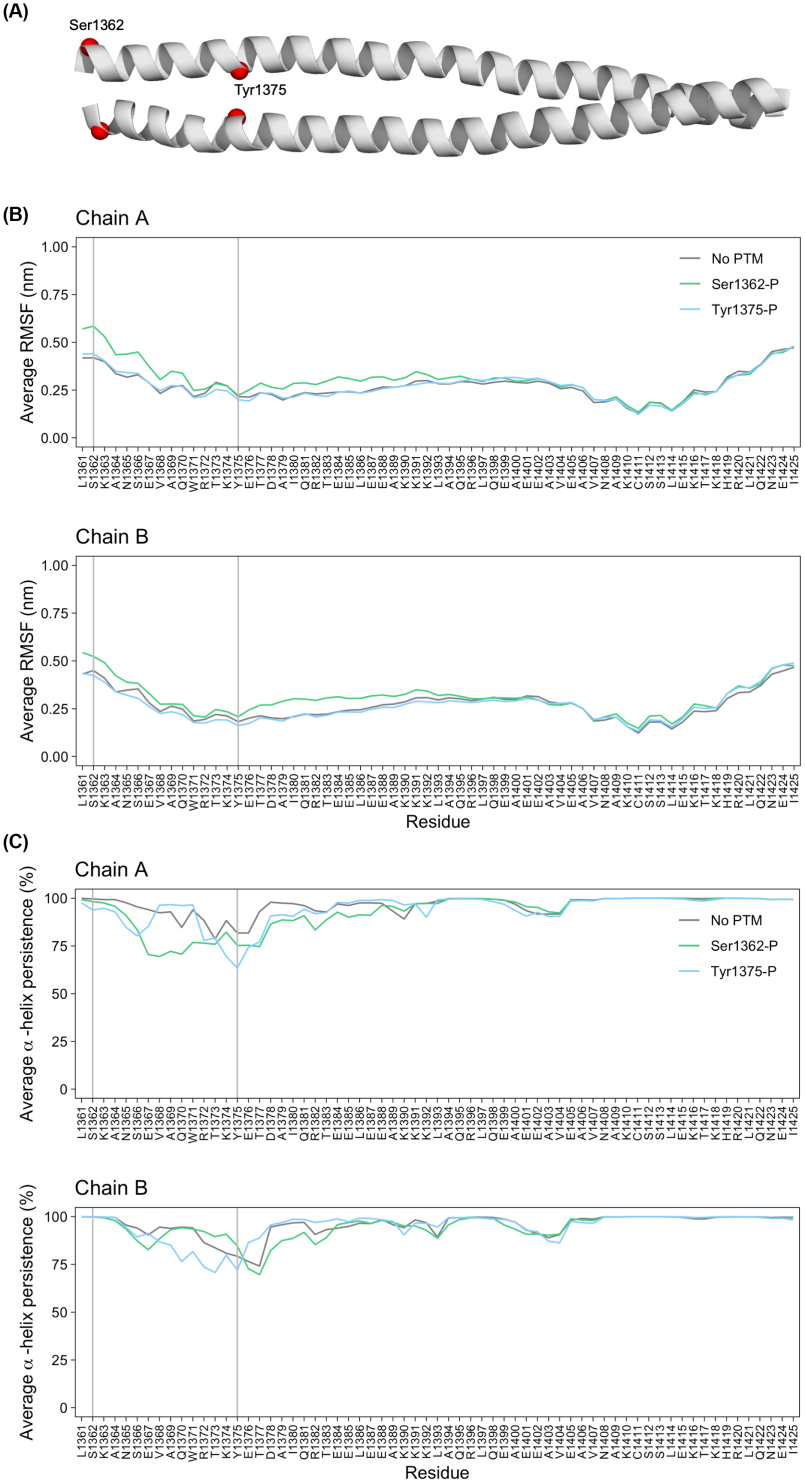
Changes in myosin flexibility and secondary structure with Ser1362‐P and Tyr1375‐P. (A) Cartoon representation of the skip 2 segment (PDB ID: 4XA3, after energy minimization). The positions of Ser1362 and Tyr1375 are each highlighted with red spheres (C_α_ atoms). (B, C) Root‐mean‐square fluctuation (RMSF) profiles (B) and α‐helix persistence (C) for molecular dynamics (MD) simulations with no post‐translational modifications (gray), with phosphorylated Ser1362 on both chains (Ser1362‐P, green) and with phosphorylated Tyr1375 on both chains (Tyr1375‐P, light blue). For each residue, values are calculated as averages over 7 MD replicas. The α‐helix persistence of a residue is calculated as the percentage of frames in which the residue is found to be part of an α‐helix. The positions of Ser1362 and Tyr1375 in the plots are highlighted with vertical lines.

To further strengthen the link between unusual PTMs and myosin behavior, we studied the impact of PTMs on myosin rod structure and dynamics. For that, we selected the two phosphorylations close to each other in an under‐studied portion of MyHC: one specific to patients with a congenital myopathy (Ser1362‐P) and one non‐specific, present in both groups of patients (Tyr1375‐P). The effects of these PTMs on the myosin rod structure and dynamics were then investigated at the atomistic level starting from the experimental structure of the skip 2 segment of the myosin rod (Figure 5A).

The impact of phosphorylation on the thermodynamic stability of the protein was initially estimated using DynaMut2.^22^ The server was used to predict the change in the free energy of unfolding (ΔΔG) induced by the phosphomimetic mutations Ser1362Glu and Tyr1375Glu, with a negative ΔΔG indicating a destabilizing mutation (Table 2). While Ser1362Glu was found to be only mildly destabilizing, the effect of Tyr1375Glu was predicted to be more pronounced. Interestingly, different values were obtained for chain A and chain B when changing one residue at a time (“single mutation” in Table 2), indicating that the environment of equivalent residues in the two chains is not the same. Moreover, for both Ser1362Glu and Tyr1375Glu, the ΔΔG obtained when changing the residue in both chains (“double mutation”) was larger than the sum of the single‐mutation values, suggesting that some cooperativity might occur.

**TABLE 2:** Predicted change in the thermodynamic stability (ΔΔG) of the myosin rod for the phosphomimetic mutations Ser1362Glu and Tyr1375Glu.

Mutation	Chain	ΔΔG—single mutation (kcal/mol)^a^	ΔΔG—double mutation (kcal/mol)^b^
Ser1362Glu	A	−0.07	−0.86
Ser1362Glu	B	−0.01	
Tyr1375Glu	A	−1.90	−3.43
Tyr1375Glu	B	−1.04	

^a^ Predicted DynaMut2 stability change when only one chain is mutated.

^b^ Predicted DynaMut2 stability change when both chains are mutated.

Molecular dynamics (MD) simulations were subsequently run to model more directly the effect of the PTMs on the protein structure and dynamics. Multiple 50‐ns replicas were run on the system with no phosphorylation (“no PTM”), with Ser1362 phosphorylated on both chains (“Ser1362‐P”) and with Tyr1375 phosphorylated on both chains (“Tyr1375‐P”). Inspection of root‐mean‐square fluctuation (RMSF) profiles for the three systems (Figure 5B and Figure S1A) shows a common shape with two minima, close to the regions where local alterations of the secondary structure were observed (Figure 5C and Figure S1B). Indeed, the α‐helical structure of the two chains was in general preserved during the simulations; however, bending and/or local unfolding was observed in some regions, leading to a decrease in their α‐helical content (persistence <100% in Figure 5C). While some variability was found across the replicas for all the systems, both PTMs tend to destabilize the secondary structure compared with the unphosphorylated system. For Ser1362‐P, this decrease in the α‐helical content is also associated with an increase in the flexibility as measured by the RMSF, in particular in the first half of each chain (green in Figure 5B).

**FIGURE 6:**
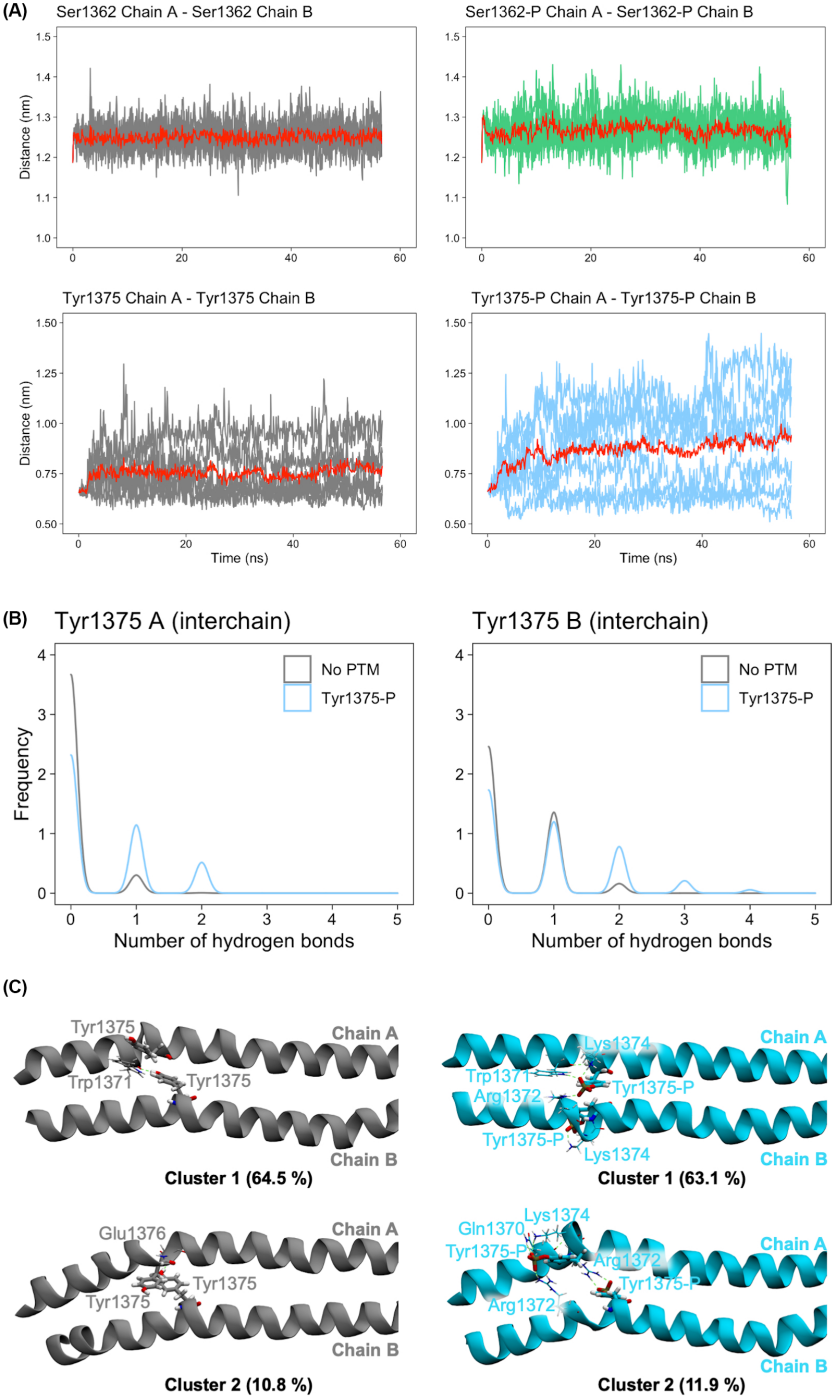
Alterations of interchain distance and interactions with Ser1362‐P and Tyr1375‐P. (A) Time evolution of the distance between the C_α_ atoms of Ser1362 (top row) and Tyr1375 (bottom row) in the two chains for no post‐translational modifications (gray), Ser1362‐P (green), and Tyr1375‐P (light blue) molecular dynamics (MD) simulations of the skip 2 segment. The average calculated over all the replicas is plotted in red. (B) Histogram of the number of interchain hydrogen bonds between Tyr1375 and any residue in the opposing chain for chain A (left plot) and chain B (right plot). Histograms show values from all the no PTM (gray) and Tyr1375‐P (light blue) MD replicas. (C) Cartoon representation of the representatives of the two most populated clusters for no PTM (left panels) and Tyr1375‐P (right panels) MD simulations. Tyr1375(‐P) residues are shown as sticks, together with the amino acids that form hydrogen bond interactions with the Tyr1375(‐P) side chains. Cluster populations are reported in brackets. Hydrogen bonds detected by VMD are shown as dashed green lines.

Analysis of interchain distances showed that for both Ser1362 and Tyr1375, phosphorylation tends to increase the distance between the backbone of the two phosphorylated residues (Figure 6A and Figure S2). This effect is more evident in Tyr1375‐P simulations, where phosphorylation also seems to promote the formation of hydrogen bonds between each phosphorylated Tyr and the opposing chain (interchain hydrogen bonds, light blue in Figure 6B). The formation of additional hydrogen bonds after phosphorylation could be the driving force for the local rearrangements observed in the in Tyr1375‐P simulations. This effect is illustrated by representative structures for the no PTM and Tyr1375‐P simulations (Figure 6C). In the no PTM structures (left), only Tyr1375 from chain B is involved in a single hydrogen bond interaction with chain A. In the Tyr1375‐P structures (right), both Tyr1375‐P residues form hydrogen bond interactions with the opposing chain, which in cluster 2 is associated with stronger distortions of the secondary structure and larger bending of the helices. No interchain hydrogen bond interactions were found for Ser1362 in either phosphorylation state. A slight increase in intrachain hydrogen bonding was observed upon phosphorylation (Figure S3), indicating that the effects of this PTM on the structure and dynamics of the protein might be mostly due to electrostatic repulsion. It is to be noted that all the present simulations were run on an isolated myosin rod segment. Effects on the dynamics of rods due to their packing in the thick filament are thus not considered. On the contrary, it is possible that the observed propensity of phosphorylation to disrupt the secondary structure of individual rods could in turn lead to destabilization of their overall packing in the filament.

## 3 DISCUSSION

Here, in the presence of myopathy‐related *RYR1* mutations, we mainly observed three additional (Thr1309‐P, Ser1362‐P, and Lys1410‐Ac on the β/slow MyHC) and six missing (Lys35‐Ac, Lys663‐Ac, Lys763‐Ac, Lys1171‐Ac, Lys1360‐Ac, and Lys1733‐Ac on the type IIa MyHC) PTMs. We also found that the ATP turnover time of myosin molecules in the disordered–relaxed state was significantly decreased. Overall, these findings suggest myosin structural and functional remodeling potentially influencing the myopathic phenotype of *RYR1* patients at the molecular level.

### 3.1 Implications of unusual post‐translational modifications in the myosin head region

One major regulator of the myosin relaxed states in cardiac and skeletal muscle is the myosin interacting head motif (IHM), which is an evolutionarily conserved structure promoting the folding back of the two myosin heads onto the filament backbone.^23^, ^24^, ^25^ Specifically, IHM involves the actin‐binding region of a blocked head and the converter region of a free head. There are intra‐molecular interactions within the blocked head (IHM priming) enabling its docking onto its own subfragment 2. There are also inter‐molecular interactions between the blocked head and the neighboring myosin rod region (IHM anchoring). Finally, there are intra‐molecular interactions between the free and blocked heads (IHM stabilization). IHM priming, anchoring, and stabilization play an essential role in lowering the myosin ATPase activity in relaxed muscle fibres. In the present study, we found that the acetylations on Lys35, Lys663, and Lys763 are missing for myopathic patients with *RYR1* mutations (Figure 2A,B). In the scientific literature, point mutations on some of these specific/partner residues induce hypertrophic cardiomyopathy^26^, ^27^, ^28^ and are pathogenic as they are predicted to trigger an alteration of the IHM. Indeed, Arg663His may affect the priming; Lys762Arg may modify the anchoring and the stabilizing; Val763Met and Val763Gly may disrupt the stabilizing; and Lys766Gln may disturb the stabilizing.^25^ Hence, we can speculate that the lack of Lys663‐Ac in the actin‐binding region and the lack of Lys763‐Ac in the converter domain^29^ may induce similar changes in priming, anchoring, and stabilizing of the IHM. These changes are likely to be subtle, though, as here, we did not observe any major shift in the super‐relaxed‐to‐disordered–relaxed state ratio, but we only found a decrease in the ATP turnover rate lifetime of myosin disordered–relaxed state of myopathic patients.

### 3.2 Impacts of aberrant PTMs in the myosin rod

Besides Lys35‐AC, Lys663‐Ac, and Lys763‐Ac, in the present study, Lys1171‐Ac, Thr1309‐P, Ser1362‐P, Lys1360‐Ac, Lys1410‐Ac, and Lys1733‐Ac were also misregulated in patients with *RYR1* mutations and congenital myopathy (Figures 1 and 2). All these are located in the under‐studied tail region, subfragment 2, or light meromyosin segment.^29^ Previous publications have highlighted that point mutations or deletions in similar regions of the MyHC molecule lead to muscle phenotype and dilated cardiomyopathy and/or skeletal myopathy,^30^, ^31^, ^32^, ^33^, ^34^, ^35^, ^36^, ^37^ including Arg1193His, Arg1193Ser, Ser1297Val, Ala1332Thr, Arg1344Trp, Glu1426Lys, Arg1434Cys, Lys1444Glu, Lys1729del, and K1784del. Additionally, abnormal PTMs such as Lys951‐Ac and Lys1195‐Ac favor molecular pathological remodeling, cardiac dysfunction, and ischaemic heart failure.^18^ In fact, the absence of Lys951‐Ac and Lys1195‐Ac disrupts electrostatic interactions, the native structure of the coiled coil, and local flexibility within the myosin tail.^18^ We suggest comparable molecular modifications here in the *RYR1* myopathic patients. This would then subtly increase the rod flexibility, which in turn would negatively adjust the orientation of myosin heads and their ability to utilize ATP or form proper IHMs. To further emphasize the potential pathogenic role of other PTMs than acetylation in the tail region, we complemented our study with MDs simulations focusing on two additional phosphorylations (Ser1362‐P and Tyr1375‐P). As our LC/MS analysis revealed that Tyr1375‐P was not specific to myopathic patients and was also present in patients with *RYR1*‐related rhabdomyolysis/myalgia and no muscle weakness, we anticipated stronger changes in myosin rod structure and dynamics for Ser1362‐P. However, both Ser1362‐P and Tyr1375‐P had pronounced destabilization effects on the tail region, indicating that particular PTMs cannot directly contribute to the development of myopathic/weakness features but may be involved in the generation of other muscle symptoms common to all the patients. Future works studying this are warranted. While in the present study, we focused our attention on phosphorylation and acetylation, it should be noted that other PTMs may be dysregulated and they may functionally disrupt MyHCs. Among these are ubiquitination, fatty acylation, O‐GlcNAcylation, and redox modifications (carbonylation, S‐nitrosylation, and S‐glutathionylation). For instance, in mouse hearts, S‐nitrosylation of myosin and myosin‐binding proteins has been proven to be critical for normal myofilament functioning.^38^

## 4 MATERIALS AND METHODS

### 4.1 Human subjects

Five patients diagnosed with congenital myopathies, five gender‐ and age‐matched control individuals with no history of neuromuscular disease, and five patients with *RYR1* mutations causing exertional rhabdomyolysis/myalgia syndrome but no muscle weakness were included in the present study. The details related to all the individuals are presented in Table 1. Note that CTL refers to controls, RYR1‐CM to *RYR1* patients with congenital myopathy, and RYR1‐RM to *RYR1* patients with rhabdomyolysis/myalgia but no muscle weakness. We obtained written informed consent from all the patients and controls. Biopsy specimens were obtained from the vastus lateralis muscle using a 5‐mm Bergstrom needle and were subsequently snap‐frozen in isopentane cooled in liquid nitrogen and stored at −80°C. Tissue storage and usage followed a local Danish ethical approval.

### 4.2 Solutions

The description of the solutions has previously been published.^40^ Briefly, the relaxing solution had 4 mM Mg‐ATP, 1 mM free Mg^2+^, 10^−6^ mM free Ca^2+^, 20 mM imidazole, 7 mM EGTA, and 14.5 mM creatine phosphate. KCl was used to adjust the pH to 7.0 and the ionic strength to 180 mM. Finally, the rigor buffer, which is essential for running Mant‐ATP chase experiments, had 120 mM K acetate, 5 mM Mg acetate, 2.5 mM K_2_HPO_4_, 50 mM MOPS, and 2 mM DTT (pH of 6.8).

### 4.3 Muscle fibre preparation

As previously detailed,^40^, ^41^ biopsy specimens were stored for 24 h at −20°C, in a skinning solution containing the relaxing solution together with glycerol (50:50 v/v). In order to facilitate the membrane permeabilization, biopsy specimens were separated into bundles and stored at 4°C in the skinning solution for an additional 24 h. Following these procedures, bundles were kept for a maximum of 1 week at −20°C in the same buffer.

### 4.4 Mant‐ATP chase experiments

On the day of testing, bundles were moved from the skinning buffer to the relaxing solution and individual myofibres were manually isolated. An explanation of our homemade chambers and our specific myofibre preparations is presented elsewhere.^42^ Briefly, our chambers were placed on a Zeiss Axio Scope A1 microscope stage where the sarcomere length of isolated muscle fibres was assessed using the brightfield mode. For the subsequent measurements, only myofibres having a sarcomere length of 2.20 μm were retained. At 25°C, each muscle fibre within a chamber was initially incubated for 5 min with a rigor buffer. The chamber was then flushed with a rigor buffer having 250 μM Mant‐ATP. Such solution was maintained in the chamber for a duration of 5 min. After completing this step, a second rigor solution containing 4 mM unlabelled ATP was introduced allowing the Mant‐ATP chase. For the acquisition, a Plan‐Apochromat 20×/0.8 objective and a Zeiss AxioCam ICm 1 camera were used. Images were recorded every 5 s for 5 min (20‐ms acquisition/exposure time using a DAPI filter set). The analysis of the fluorescence decay consisted of taking three regions for each muscle fibre for each image, using the ROI manager in ImageJ as previously published.^42^ After background fluorescence intensity subtraction, data were fit to an unconstrained double exponential decay using GraphPad Prism 9.0:

$$\begin{array}{c} \text{Normalized Fluorescence}=\\ 1-\text{P1}\;\left(1-\exp^{\left(-t/\text{T1}\right)}\right)-\text{P2}\;\left(1-\exp^{\left(-t/\text{T2}\right)}\right), \end{array}$$

where P1 is the amplitude of the initial rapid decay (related to the disordered–relaxed state) with T1 as the time constant for this decay. P2 is the slower second decay (related to the super‐relaxed state) with its associated time constant T2.^42^

### 4.5 Immunofluorescence staining and imaging

For the human Mant‐ATP chase experiments, we defined the MyHC subtypes using immunofluorescence staining as previously detailed.^42^ An anti‐β‐cardiac/skeletal slow myosin heavy antibody (IgG1, A4.951, sc‐53 090 from Santa Cruz Biotechnology, dilution: 1:50) together with a goat anti‐mouse IgG1 Alexa 555 (from Thermo Scientific, dilution 1:1000) was used.^40^, ^41^

### 4.6 Myosin band excision, in‐gel digest, and LC/MS

For the detailed description of protein separation (SDS–PAGE), MyHC band excision, tryptic in‐gel digest, peptide separation, and sequencing, please see Ref.^43^ Raw mass spectrometry data were analyzed with MaxQuant (v1.6.15.0). A summary of all the phosphorylation (STY) and acetylation (K) sites found (with a probability >0.9) is shown in Table S1. The mass spectrometry proteomics data have been deposited to the ProteomeXchange Consortium via the PRIDE partner repository with the dataset identifier PXD041906.^44^

### 4.7 Molecular modeling and simulation

The X‐ray structure of the rod region surrounding skip 2 in human MYH7 (PDB ID: 4XA3–^45^) was used to model the effect of Ser1362 and Tyr1375 phosphorylation on the protein structure and dynamics. The whole structure was used for the calculations, but analyses focused on the *MYH7* (β/slow MyHC) portion of the chimeric construct (residues 1361–1425). Short missing segments in the non‐*MYH7* part of the structure were modeled with ModLoop.^46^ The effect of the phosphomimetic mutations Ser1362Glu and Tyr1375Glu on the thermodynamic stability was predicted with DynaMut2.^22^

MD simulations were run on three different phosphorylation states: no PTMs, phosphorylated Ser1362 on both chains (Ser1362‐P), and phosphorylated Tyr1375 on both chains (Tyr1375‐P). Phosphate groups were added at the relevant positions of the coiled coil using the Schrödinger Maestro visualization software (Schrodinger suite, release 2021‐2).

All simulations were performed using GROMACS 2020 and the Amber99SB*‐ILDN force field.^47^ Amber force‐field parameters were also used for the phosphorylated residues.^48^ The systems were solvated with a truncated octahedral box of TIP3P water molecules. Ionizable residues were set to their standard protonation state at pH 7, and the systems were neutralized with the addition of counterions for an overall ionic strength of 50 mM. The total number of atoms was ~612 075. Periodic boundary conditions with a minimum distance of 1.2 nm between the protein and the walls of the box were applied. The particle mesh Ewald method was used for electrostatic interactions. A 0.9‐nm cutoff was used for direct space sums and for van der Waals interactions. The time step was set to 2 fs. Energy minimization, equilibration, and production were run following the simulation parameters and protocol used in previous simulations of the myosin motor domain.^49^ A total of 7 replicas were run for each phosphorylation state, with production runs of 50 ns (350 ns per state).

The flexibility of each system was measured by calculating the RMSF of the C_α_ atom coordinates saved every 1 ps (production only). The *MYH7* region of the construct was used both for the fitting of the frames and for the calculation of the RMSF values. Hydrogen bonds were analyzed with the H‐bond plugin of VMD on frames saved every 100 ps,^50^ using a threshold of 0.35 nm on the donor–acceptor distance and of 30° on the hydrogen–donor–acceptor angle. The time evolution of the protein secondary structure was calculated using the DSSP tool as implemented in GROMACS on production trajectories saved every 1 ps.^51^ For each phosphorylation state, a cluster analysis was performed on the concatenated production replicas (structures were sampled every 100 ps) using the GROMOS method implemented in GROMACS. A cutoff of 0.36 nm was used on the distance between frames, measured as the root‐mean‐square deviation (RMSD) calculated over the C_α_ atoms of the *MYH7* region.

### 4.8 Statistical analysis

Data are shown as means ± standard deviations. Graphs were generated in GraphPad Prism v9. Statistical significance was set to *p* < 0.05. *t* tests with Welsh's corrections or one‐ and two‐way ANOVAs were run to compare groups.^40^

## 5 CONCLUSION

Altogether, as the regulation of PTMs on both β/slow and type IIa MyHC molecules is a highly conserved process in skeletal muscle, the multiple phosphorylation or acetylation changes we observed between myopathic patients and controls may trigger the decreased ATP turnover time of relaxed myosin molecules disturbing the energy demand of skeletal muscle. Future studies focusing on the latter are required. Nevertheless, as muscle, in general, supplies a large amount of the basal energy requirements,^7^ we can anticipate that our findings would promote a metabolic hyperactivation consisting of a shift away from glycolysis toward oxidative phosphorylation.^39^

## AUTHOR CONTRIBUTIONS

**Alexander Sonne**: Conceptualization; Investigation; Writing—original draft; Writing—review & editing; Formal analysis; Methodology. **Anna Katarina Antonovic**: Conceptualization; Investigation; Writing—original draft; Writing—review & editing; Formal analysis; Methodology. **Elise Melhedegaard**: Investigation; Writing—review & editing; Formal analysis; Methodology. **Fariha Akter**: Investigation; Writing—review & editing; Formal analysis; Methodology. **Jesper L. Andersen**: Investigation; Writing—review & editing; Formal analysis. **Heinz Jungbluth**: Conceptualization; Writing—original draft; Writing—review & editing. **Nanna Witting**: Investigation; Writing—review & editing; Formal analysis. **John Vissing**: Investigation; Writing—review & editing; Formal analysis. **Edmar Zanoteli**: Investigation; Writing—review & editing; Formal analysis. **Arianna Fornili**: Conceptualization; Investigation; Writing—original draft; Writing—review & editing; Supervision; Funding acquisition; Data curation. **Julien Ochala**: Conceptualization; Investigation; Funding acquisition; Writing—original draft; Writing—review & editing; Data curation; Supervision; Project administration.

## CONFLICT OF INTEREST STATEMENT

The authors declare no conflicts of interest.

## Supporting information


Data S1.
