# Supplementary material for: Abnormal myosin post‐translational modifications and ATP turnover time associated with human congenital myopathy‐related RYR1 mutations
Source: Acta Physiol (Oxf). 2023 Aug 21;239(2):e14035. doi: 10.1111/apha.14035 (PMC10909445; doi:10.1111/apha.14035)
Supplement: Supplementary file 1 — Data S1. [file APHA-239-e14035-s001.docx]

**Abnormal myosin post-translational modifications and ATP turnover time associated with congenital myopathy-related *RYR1* mutations**

Alexander Sonne^1^, Anna Katarina Antonovic^2^, Elise Melhedegaard^1^, Fariha Akter^2^, Jesper L. Andersen^3,4^, Heinz Jungbluth^5,6^, Nanna Witting^7^, John Vissing^7^, Edmar Zanoteli^8^, Arianna Fornili^2^, Julien Ochala^1^*

1. Department of Biomedical Sciences, Faculty of Health and Medical Sciences, University of Copenhagen, Copenhagen, Denmark
2. Department of Chemistry, School of Physical and Chemical Sciences, Queen Mary University of London, London, UK
3. Institute of Sports Medicine Copenhagen, Department of Orthopaedic Surgery, Copenhagen University Hospital, Bispebjerg and Frederiksberg, Copenhagen, Denmark
4. Center for Healthy Aging, Department of Clinical Medicine, University of Copenhagen, Denmark
5. Department of Paediatric Neurology, Evelina London Children's Hospital, London, UK
6. Randall Centre for Cell and Molecular Biophysics, Muscle Signalling Section, Faculty of Life Sciences and Medicine, King's College London, London, UK
7. Copenhagen Neuromuscular Center, Department of Neurology, University of Copenhagen, Copenhagen, Denmark
8. Universidade de São Paulo, Faculdade de Medicina, Hospital das Clínicas, Departamento de Neurologia, São Paulo, SP, Brazil

*Corresponding author: [julien.ochala@sund.ku.dk](mailto:julien.ochala@sund.ku.dk)

**Supplementary Information**


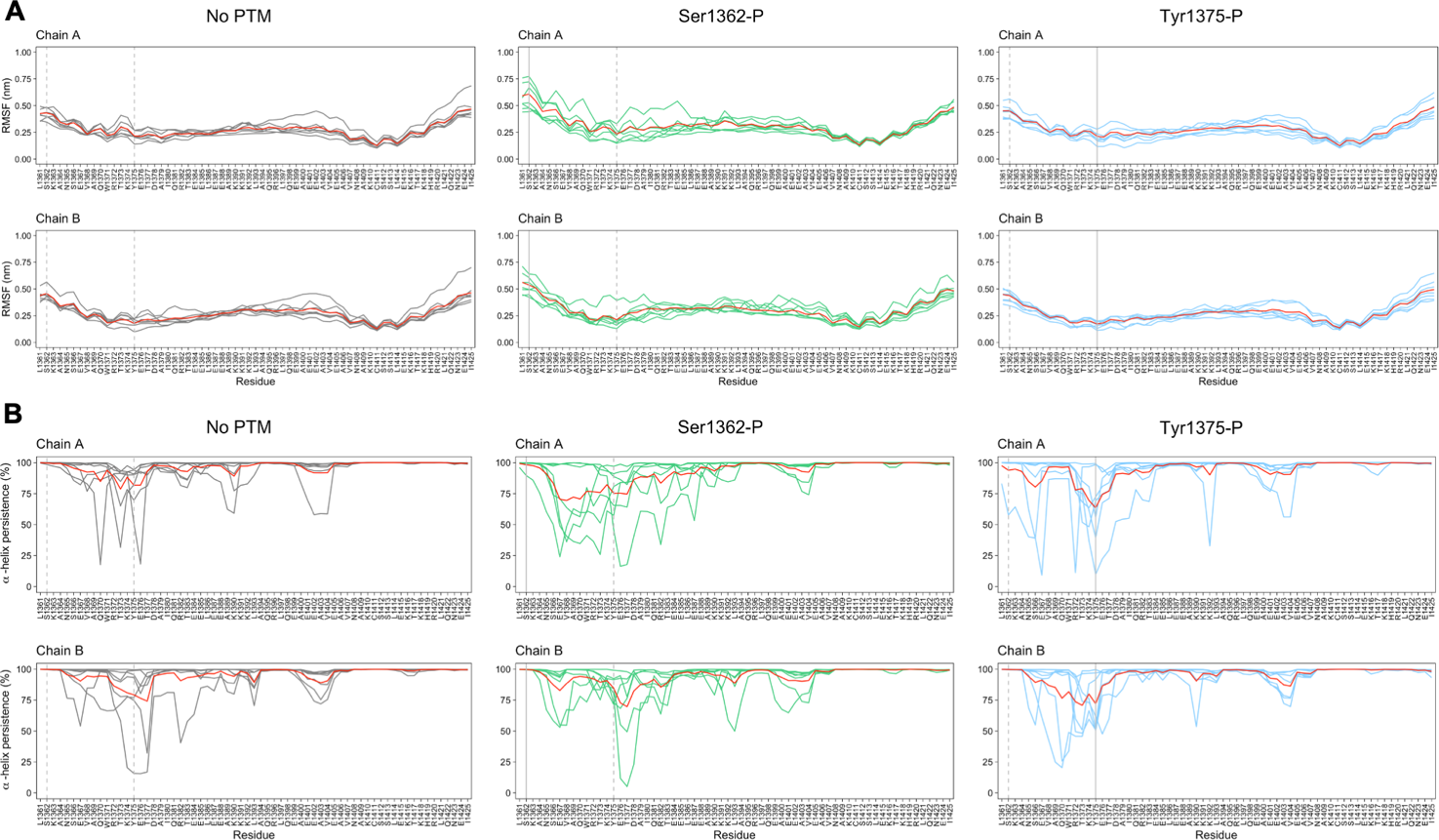


**Figure S1: Effect of PTMs on myosin flexibility and secondary structure.**

Root mean square fluctuation (RMSF) profiles (A) and α-helix persistence (B) for MD simulations (all replicas) of the skip 2 segment with no PTMs (grey), with phosphorylated Ser1362 on both chains (Ser1362-P, green) and with phosphorylated Tyr1375 on both chains (Tyr1375-P, light blue). The average calculated over all the replicas is plotted in red. The positions of Ser1362 and Tyr1375 in the plots are each highlighted with vertical lines (the line is dotted if the residue is not phosphorylated).


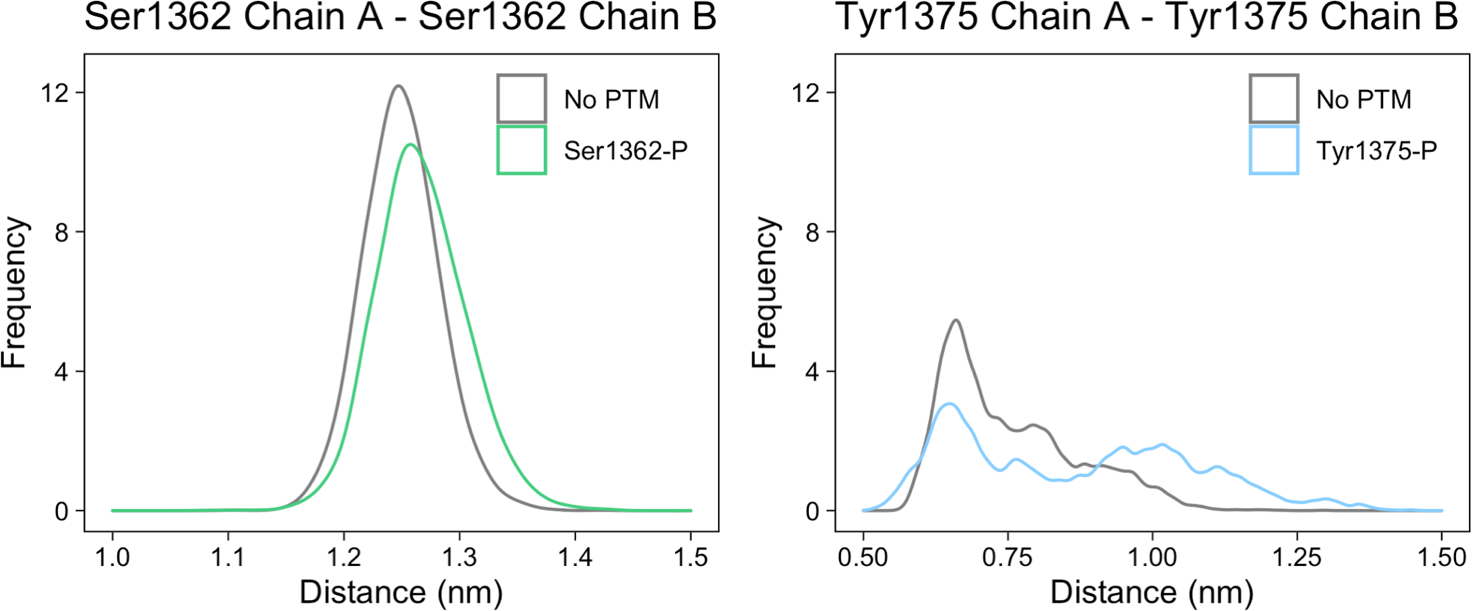


**Figure S2: Effect of PTMs on interchain distance.**

Histograms of the distance between the C_α_ atoms of Ser1362 (left) and Tyr1375 (right) in the two chains for no PTM (grey), Ser1362-P (green) and Tyr1375-P (light blue) MD simulations of the skip 2 segment. Cumulative values from all the replicas are reported for each phosphorylation state.


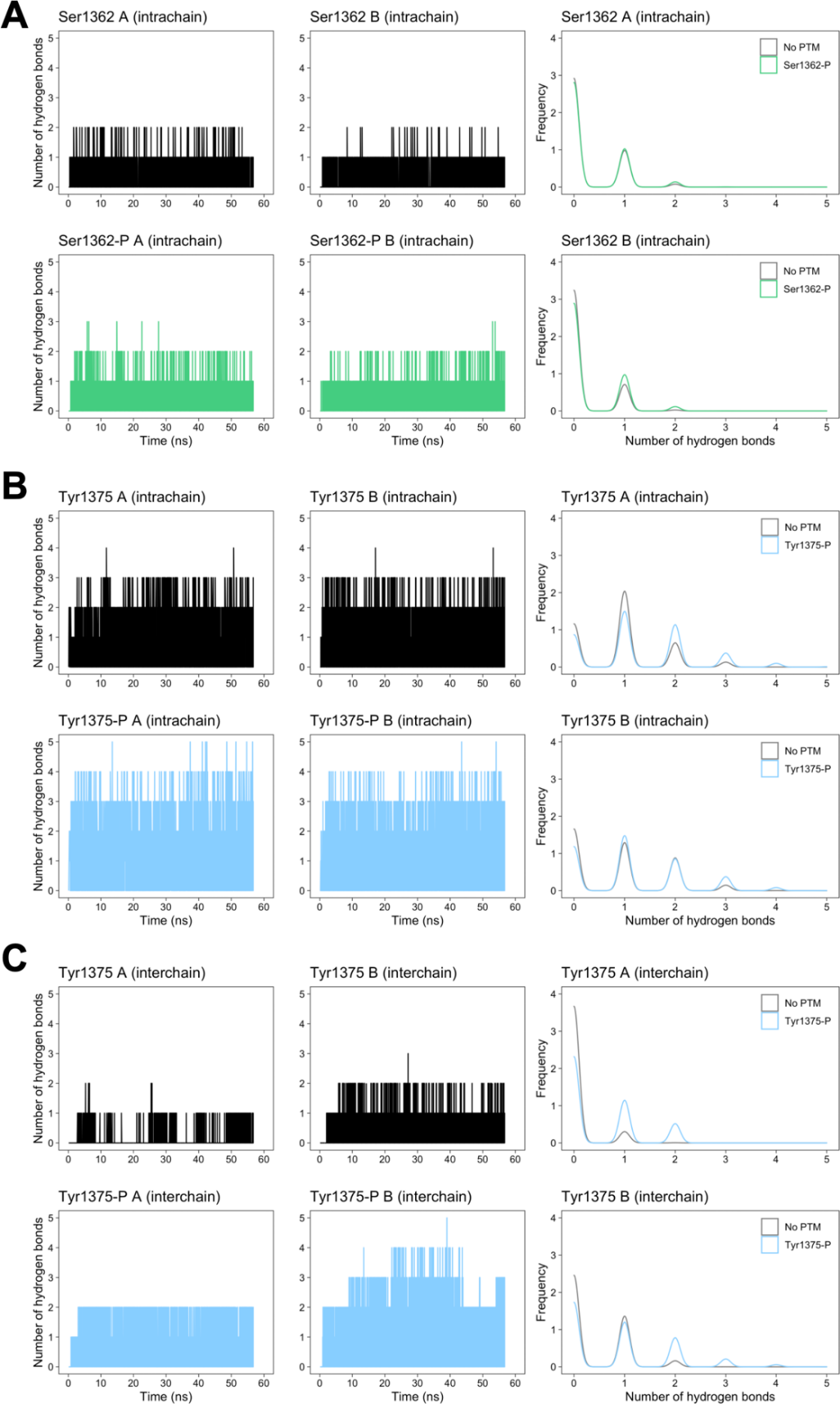


**Figure S3: Effect of PTMs on hydrogen bond interactions.**

Time evolution of the number of intrachain hydrogen bonds involving Ser1372 (A) and Tyr1375 (B) and time evolution of the number of interchain hydrogen bonds involving Tyr1375 (C), together with the corresponding histograms (right panels). Cumulative values from all the replicas of no PTM (grey), Ser1362-P (green) and Tyr1375-P (light blue) MD simulations are reported.

**Table S1: Summary of all the phosphorylation and acetylation sites found (with a probability >0.9).**

The whole list and mass spectrometry proteomics data have been deposited to the ProteomeXchange Consortium via the PRIDE partner repository with the dataset identifier PXD041906. MyHC refers to myosin heavy chain.

| **β/slow MyHC**  **P12883 - MYH7_HUMAN** | | **Type IIa MyHC**  **Q9UKX2 - MYH2_HUMAN** | |
| --- | --- | --- | --- |
| **Phosphorylation** | **Acetylation** | **Phosphorylation** | **Acetylation** |
| Ser1102  Ser1275  Thr1307  Tyr1308  Thr1309  Ser1362  Tyr1375  Ser1478  Ser1645  Ser1718  Thr1721 | Lys34  Lys43  Lys72  Lys757  Lys847  Lys920  Lys976  Lys1073  Lys1165  Lys1173  Lys1305  Lys1326  Lys1410  Lys1444  Lys1499  Lys1528  Lys1579  Lys1617  Lys1757  Lys1770  Lys1784  Lys1874 | Tyr413  Ser625  Ser1143  Ser1146  Ser1153  Ser1305  Ser1308  Tyr1381  Ser1482  Thr1724  Thr1727  Ser1728  Thr1732 | Lys35  Lys73  Lys87  Lys663  Lys763  Lys853  Lys859  Lys882  Lys982  Lys997  Lys1079  Lys1171  Lys1179  Lys1231  Lys1360  Lys1623  Lys1674  Lys1733  Lys1735  Lys1763  Lys1776 |
|  |  |  |  |
